# Predictive assessment in pharmacogenetics of Glutathione S-transferases genes on efficacy of platinum-based chemotherapy in non-small cell lung cancer patients

**DOI:** 10.1038/s41598-017-02833-7

**Published:** 2017-06-01

**Authors:** Huan Ye, Meiqin Shao, Xiaohong Shi, Lifeng Wu, Bing Xu, Qiang Qu, Jian Qu

**Affiliations:** 1 Department of Respiratory, Wenzhou People’s Hospital, Wenzhou, 325000 People’s Republic of China; 2 Department of Pharmacy, Xiangya Hospital, Central South University, Changsha, 410078 People’s Republic of China; 3 Department of Pharmacy, the Second Xiangya Hospital, Central South University; Institute of Clinical Pharmacy, Central South University, Changsha, 410011 People’s Republic of China

## Abstract

The influences of glutathione s-transferase P1, M1, and T1 variants on the efficacy of platinum-based chemotherapy in non-small cell lung cancer (NSCLC) patients were inconsistent in previous studies. Our meta-analysis enrolled 31 publications including 5712 patients and provided more convincing and reliable conclusions. Results showed that *GSTP1* IIe105Val IIe/Val and Val/Val Asian patients were more likely to have better response rates compared to IIe/IIe patients (odds ratio (OR) = 1.592, 95% confidence intervals (CIs), 1.087–2.332, *P* = 0.017). The Asian patients bearing the favorable *GSTM1* null genotype were more likely to have better response rates to platinum-based chemotherapy compared to those patients with the unfavorable *GSTM1* present genotype (OR = 1.493 (1.192–1.870), *P* < 0.001). Caucasian lung cancer patients bearing *GSTT1* null genotype might be more closely associated with shorter survival time and higher risks of death than the *GSTT1* present patients (hazard ratio (HR) = 1.423, CI = 1.084–1.869, *P* = 0.011). Our meta-analysis suggested that the *GSTP1* IIe105Val, *GSTM1* and *GSTT1* null variants might be predictive factors for the efficacy of platinum-based chemotherapy to NSCLC patients. The use of *GSTP1* IIe105Val, *GSTM1* and *GSTT1* null polymorphisms as predictive factors of efficacy of personalized platinum-based chemotherapy to NSCLC patients requires further verification with multi-center, multi-ethnic and large-sample-size pharmacogenetic studies.

## Introduction

Lung cancer is the most common cancer worldwide and the most common causes of cancer death are cancers of the lung and bronchus in both man and woman^1, 2^. About 80% of lung cancer was non-small cell lung cancer (NSCLC), which is diagnosed at an advanced stage with approximate 15% of the 5-year survival rate^3^. Current studies showed that the prognosis of NSCLC was contributed to patients’ clinical status and genetic factors such as TNM staging, surgery, chemotherapy drugs, genetic heterogeneity including *EGFR*, *KRAS*, *PIK3CA*, *ALK et al*.^4, 5^. Chemotherapy is the main conventional and useful therapeutic method for advanced and metastatic tumors^6, 7^. NSCLC accounts for approximately 70% of patients harboring advanced stages at the time of diagnosis and chemotherapy is the important treatment strategy for NSCLC patients^8, 9^. Platinum-based chemotherapy is one of effective treatments in advanced lung cancer patients, which could improve the survival of patients^10, 11^. The efficacy of platinum-based chemotherapy was individual differences among patients^12, 13^. Scientists have spared no efforts to search for relevant therapeutic and prognostic biomarkers to improve the accuracy and sensitivity of prognostic and predictive assessment in NSCLC patients. However, there is still a lack of perfect biomarkers and clinical practice.

It is well known that platinum acts through the formation of bulky intrastrand and interstrand DNA adducts that inhibit DNA synthesis and transcription^14^. Moreover, studies have suggested that the resistant mechanisms of platinum may be via the inactivation of platinum compounds through the glutathione metabolic pathway and via the increase of the DNA repair capacity and of the tolerance to DNA damage^14–16^. Glutathione S-transferases (GSTs) are a series of phase II metabolic enzymes, which are involved in the platinum detoxification^17^. GSTM1, GSTP1 and GSTT1 are the most important GSTs enzymes^18^. Evidences showed that the variants of *GSTP1* (rs1695, Ile105Val), *GSTM1* (null/present) and *GSTT1* (null/present) may be involved in the platinum-based treatment, but the results were not consistent^14–16, 19–39^.

Meta-analysis rather than a single study can provide more comprehensive and compelling conclusions by systematically summarizing and analyzing previous data^6^. There were two meta-analyses which reported the inconsistent results for evaluating the associations between *GSTP1* and *GSTM1* polymorphisms and response to platinum-based chemotherapy in lung cancer^17, 40^. These two meta-analyses have not enrolled update studies and just analyzed a few studies, and thus may have biased conclusions. Moreover, up to now there have been no meta-analysis concerning in *GSTT1* deletion polymorphism and the response to platinum-based chemotherapy in lung cancer. Therefore, after combining all available data and derived more precise and comprehensive assessment, we have updated this system review and meta-analysis to find out the reliable associations of *GSTP1* (Ile105Val), *GSTM1* (null/present) and *GSTT1* (null/present) variants with the efficacy and clinical outcomes of NSCLC patients treated with platinum-based chemotherapy.

## Results

### Study selection and characteristics of studies

**Figure 1:**
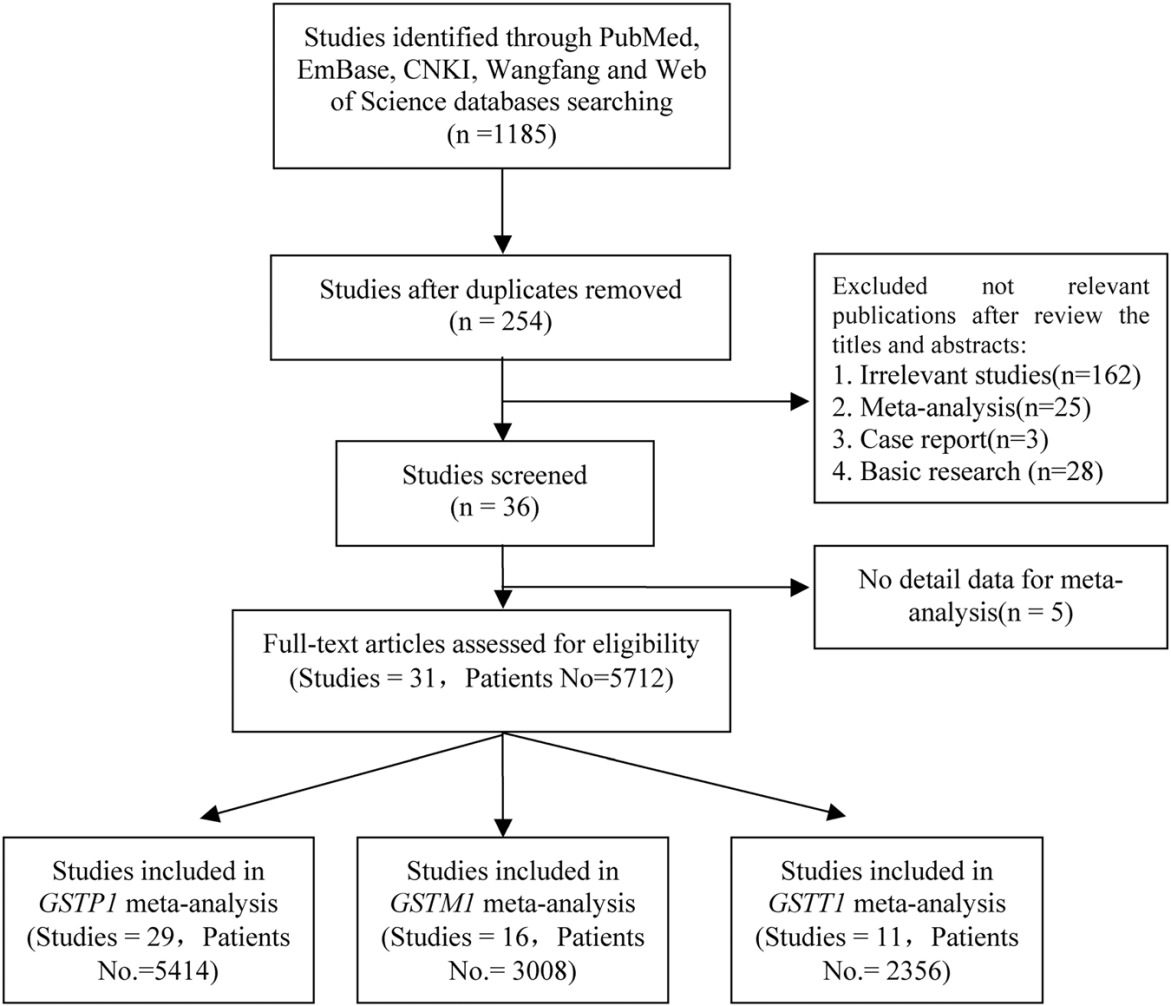
Procedure of article selection.

A total of 254 publications were found after excluding the duplication studies from 1185 publications. We excluded 162 irrelevant studies, 25 meta-analyses, 3 case reports, and 28 basic studies. Thirty-six studies were included for further review. After excluding no-detail data for meta-analysis, 31 papers including 5712 patients were enrolled in the final analysis. Among them, 29 studies were involved in *GSTP1* Ile105Val; 16 studies were involved in *GSTM1* (null/present); and 11 studies were involved in *GSTT1* (null/present). The enrolled details and CONSORT diagram are shown in Fig. 1. The characteristics of first author name, publishing year, country, ethnicity, age, smoking percentage, clinical stage, method of detect polymorphisms, quality score (QS), and the number of patients were shown in Table 1. Twenty-two of the included studies were conducted on Asian patients and 9 were on Caucasian patients. The information of objective response rate (ORR), overall survival (OS) and hazard ratios (HRs), median survival time (MST), the median time to progression (TTP) and the median progression-free survival (PFS) in each study are shown in Tables 2 and 3.

**Table 1:** Characteristics of studies included in meta-analysis.

Author	Year	Country	Ethnicity	Patients numbers	Age (year)	Smoking	Clinical stage	Evaluation criterion	Outcomes	Genotyping method	Genes	QS
L. Bu *et al*.^19^	2016	China	Asian	141	55.95 ± 7.83	65.96%	III-IV	RECIST	ORR/OS	PCR-RFLP	GSTP1	14
Jia W. *et al*.^20^	2016	China	Asian	265	57.50 ± 11.25	44.26%	IIIA, IIIB, IV.	RECIST	ORR/OS	PCR-RFLP	GSTP1, GSTM1, GSTT1	15
Chen J.B. *et al*.^21^	2016	China	Asian	284	63.60 ± 11.65	60.21%	IIIA, IIIB, IV.	RECIST	OS/PFS	DNA pyrosequencing	GSTP1, GSTM1, GSTT1	22
Xiao H.L. *et al*.^22^	2016	China	Asian	262	58.42 ± 8.50	64.12%	IIIA, IIIB, IV	RECIST	ORR/OS/MST	PCR-RFLP	GSTP1, GSTM1, GSTT1	18
Liu K. *et al*.^23^	2015	China	Asian	308	66.12 ± 10.32	62.98%	IIIA, IIIB, IV	RECIST	ORR/OS	PCR-RFLP	GSTP1, GSTM1, GSTT1	15
Zhao R. *et al*.^24^	2015	China	Asian	206	56.07 ± 8.85	65.53%	III-IV	RECIST	ORR/OS/MST	PCR-RFLP	GSTP1	18
Wu G. *et al*.^25^	2015	China	Asian	282	59.15 ± 10.50	68.44%	IIIA, IIIB, IV	RECIST	ORR/OS	PCR-RFLP	GSTP1, GSTM1, GSTT1	15
Liu J.Y. *et al*.^26^	2015	China	Asian	322	62.5 ± 9.5	43.48%	IIIB, IV	RECIST	ORR/OS	PCR-RFLP	GSTP1	16
Han B. *et al*.^27^	2015	China	Asian	325	57.6 ± 12.4	68.31%	IIIB, IV	RECIST	ORR/OS/MST	PCR-RFLP	GSTP1	19
Deng J.H. *et al*.^16^	2015	China	Asian	97	NR	40.20%	IIIB, IV	RECIST	ORR/DCR/PFS	DNA pyrosequencing	GSTP1	15
Yuan Z.J. *et al*.^28^	2015	China	Asian	47	NR	NR	III-IV	RECIST	ORR	Sanger sequencing	GSTP1	13
Li Q.Y. *et al*.^57^	2014	China	Asian	89	60.73 ± 10.857	NR	III-IV	RECIST	ORR	Direct sequencing	GSTP1, GSTM1	12
Lv H. *et al*.^29^	2014	China	Asian	91	59 (34–80)	NR	III-IV	WHO	ORR/Medium TTP	TaqMan-MGB	GSTP1	15
Ruano-Ravina A. *et al*.^37^	2014	Spain	Caucasian	132	66 (38–87)	NR	I-IV	NR	OS/MST	PCR-RFLP	GSTP1, GSTM1, GSTT1	14
Li W. *et al*.^38^	2012	China	Asian	217	58.98 (24–83)	55.80%	III-IV	NR	ORR/OS	PCR-RFLP	GSTM1	12
Ke H.G. *et al*.^30^	2012	China	Asian	460	55 (32–79)	67.30%	III-IV	NR	OS	PCR- CTPP	GSTP1	14
Zhang Y.P. *et al*.^58^	2012	China	Asian	62	58 (37–72)	NR	III-IV	RECIST	ORR	TaqMan PCR	GSTP1	15
Joerger M. *et al*.^31^	2012	Switzerland	Caucasian	146	59.7 (37–79)	83%	IIIB/IV	RECIST	ORR/OS/PFS	DNA sequencing	GSTP1, GSTM1	20
Zhou F. *et al*.^59^	2011	China	Asian	94	NR	NR	IIIB, IV	RECIST	ORR	Direct sequencing	GSTP1	12
Zhou Fei *et al*.^14^	2011	China	Asian	111	57 (42–71)	NR	IV	RECIST	TPP/ORR	DNA sequencing	GSTP1	16
Sun N. *et al*.^33^	2010	China	Asian	113	59.6 (34–84)	NR	IIIA–IV	WHO	ORR	Gene-chip	GSTP1	16
Ada A.O. *et al*.^32^	2010	Turkey	Caucasian	138	56 (34–75)	90.60%	III, IV	WHO	OS	PCR-RFLP	GSTP1, GSTM1, GSTT1	15
Yue Z. *et al*.^60^	2009	China	Asian	102	61 (27–78)	NR	III-IV	WHO	ORR	PCR-RFLP	GSTP1	13
Kalikaki A. *et al*.^34^	2009	Greece	Caucasian	119	61 (39–85)	NR	IIIA, IIIB, IV	RECIST	ORR/OS/MST	PCR-RFLP	GSTP1, GSTM1, GSTT1	17
Li W. *et al*.^39^	2008	China	Asian	141	—	56%	III-IV	RECIST	ORR	PCR-RFLP	GSTP1, GSTM1	11
Sreeja L. *et al*.^49^	2008	India	Caucasian	211	57.82 ± 11.74	68.20%	I-IV	NR	OS	Multiplex PCR	GSTP1, GSTM1, GSTT1	12
Mao Y. *et al*.^61^	2007	China	Asian	59	45 (18–65)	NR	IIIB, IV	NR	ORR	PCR-LDR	GSTP1, GSTM1	10
Gonlugur U. *et al*.^62^	2006	Turkey	Caucasian	81	60 (40–78)	88%	I-IV	NR	OS	PCR-RFLP	GSTM1, GSTT1	10
Booton R. *et al*.^35^	2006	United Kingdom	Caucasian	108	NR	NR	III-IV	RECIST	ORR/OS/MST	Direct sequencing	GSTP1	19
Lu C. *et al*.^36^	2006	USA	Caucasian	425	NR	89.60%	III, IV	RECIST	OS	PCR-RFLP	GSTP1	15
Sweeney C. *et al*.^15^	2003	USA	Caucasian	274	62 (28–74)	NR	III-IV	NR	OS	PCR-RFLP	GSTP1, GSTM1, GSTT1	12

NR: not reported; QS, quality score; HR: hazard ratio; ORR: objective response rate; OS, overall survival (months); PFS, progression-free survival (months); MST, median survival time (months); TTP, time to progression (months); PCR, polymerase chain reaction; PCR-RFLP, PCR-restriction fragment length polymorphism; RECIST, Response Evaluation Criteria in Solid Tumors; WHO, World Health Organization; PCR-LDR, PCR-ligase detection reaction; PCR-CTPP, duplex PCR with the confronting-two-pair primer; Sequenome MS-based genotyping assay, sequenome mass spectrometry-based genotyping assay; PCR-CTTP, PCR with confronting two-pair primers.

**Table 2:** Association between the *GSTP1* IIe105Val polymorphism and objective response rate, median survival time, median time to progression and median progression-free survival of platinum-based chemotherapy in NSCLC patients.

GSTP1 (IIe105Val)							
Author	Year	ORR (Good + Poor)			MST/Survival time (HR)		
		IIe/IIe	IIe/Val	Val/Val	IIe/IIe	IIe/Val	Val/Val
L. Bu	2016	66 (28 + 38)	63 (34 + 29)	12 (9 + 3)	Reference	0.83 (0.26–2.62)	0.07 (0.01–0.34)
Jia W.	2016	101 (21 + 80)	105 (28 + 77)	38 (16 + 22)	Reference	1.38 (0.69–2.80)	2.77 (1.14–6.64)
Chen J.B.	2016				21.85	25.32	27.4
					Reference	0.52 (0.29–0.92)	0.37 (0.17–0.79)
Xiao H.L.	2016				16.62	16.91	17.32
		118 (62 + 56)	66 (36 + 30)	78 (45 + 33)	Reference	0.99 (0.5–1.98)	0.58 (0.31–1.08)
Liu K.	2015				30.25 ± 2.06	37.63 ± 2.01	39.84 ± 3.36
		101 (82 + 19)	116 (71 + 45)	45 (18 + 27)	Reference	0.51 (0.28–0.94)	0.35 (0.16–0.78)
Zhao R.	2015				19.43 ± 1.62	21.27 ± 1.49	42.76 ± 4.28
		91 (36 + 55)	94 (54 + 40)	21 (16 + 5)	Reference	0.65 (0.25–1.66)	0.05 (0.01–0.18)
Wu G.	2015	120 (42 + 78)	89 (41 + 48)	74 (41 + 34)	Reference	0.58 (0.31–1.07)	0.48 (0.25–0.93)
Liu J.Y.	2015	181 (123 + 58)	114 (69 + 45)	27 (6 + 21)	Reference	1.58 (0.94–2.66)	4.35 (1.40–17.92)
Han B.	2015				22.2	27.1	34.5
		148 (88 + 60)	149 (115 + 34)	28 (25 + 3)	Reference	0.75 (0.46–1.22)	0.36 (0.11–0.98)
Deng J.H.	2015						
		70 (24 + 46)	27 (4 + 23)				
Yuan Z.J.	2015	30 (16 + 14)	15 (7 + 8)	2 (1 + 1)			
Lv H.	2014	47 (10 + 37)	44 (24 + 20)				
Ke H.G.	2012				Reference	1.39 (0.95–2.03)	1.89 (1.10–3.17)
Joerger M.	2012				12.4 (6.6–15.9)	9.8 (8.2–11.0)	9.1 (1.6–16.2)
		55 (20 + 35)	60 (18 + 42)	17 (3 + 14)	Reference	1.34 (0.89–2.02)	1.32 (0.72–2.42)
Zhou F.	2011						
		63 (13 + 50)	48 (22 + 26)				
Ada A.O.	2010				Reference	1.44 (0.78–2.63)	
Sun N.	2010	71 (13 + 58)	38 (15 + 23)	4 (2 + 2)			
Kalikaki A.	2009				10.2 (8.2–12.2)	12.9 (10.9–15.0)	
		74 (25 + 48)	42 (12 + 30)		Reference	0.5 (0.33–0.84)	
Sreeja L.	2008				23	20	12
					Reference	1.5 (0.831–2.886)	1.4 (0.619–3.522)
Booton R.	2006				9.3 (7.5–11.0)	10.8 (5.6–15.9)	7.7 (6.5–8.9)
		38 (13 + 25)	32 (12 + 20)	16 (4 + 12)	Reference	0.83 (0.44–1.58)	1.14 (0.52–2.50)
Lu C.	2006				Reference	1.24 (0.97–1.58)	0.88 (0.60–1.30)
Zhang Y.P.	2012	42 (7 + 35)	20 (9 + 11)				
Yue Z.	2009	37 (15 + 22)	17 (11 + 6)	2 (2 + 0)			
Li Q.Y.	2014	62 (18 + 44)	27 (8 + 19)				
Zhou F.	2011	49 (10 + 39)	45 (20 + 25)				
Mao Y.	2007	32 (10 + 22)	20 (8 + 12)	7 (4 + 3)			
Sweeney C.	2003				Reference	0.85 (0.57–1.27)	1.55 (0.84–2.87)
**Author**	**Year**	**PFS/HR**			**Median TTP/HR**		
		**IIe/IIe**	**IIe/Val**	**Val/Val**	**IIe/IIe**	**IIe/Val**	**Val/Val**
Chen J.B.	2016	16.6	21.65	22.52			
		Reference	0.37 (0.18–0.74)	0.15 (0.06–0.35)			
Deng J.H.	2015	198 (158.2–237.8)	171 (82.8–259.2)				
		Reference	1.639 (1.014–2.650)				
Lv H.	2014				5.23 (4.459–6.009)	9.56 (8.763–10.350)	
Joerger M.	2012	7.0 (4.5–8.2]	5.3 (4.2–6.3)	6.0 (4.2–9.3)			
		Reference	1.34 (0.89–2.02)	1.32 (0.72–2.42)			
Zhou F.	2011				6.5 (5.785–7.215)	9.0 (8.365–9.635)	
					1.852 (1.185–2.893)	Reference	

HR: hazard ratio; MST, median survival time (months); TTP, time to progression (months); PFS, progression-free survival (months); ORR: objective response rate.

**Table 3:** Association between the *GSTM1* and *GSTT1* polymorphisms and objective response rate, median survival time, median time to progression and median progression-free survival of platinum-based chemotherapy in NSCLC patients.

GSTM1							
Author	Year	ORR (Good + Poor)		MST/HR		PFS/HR	
		Present	Null	Present	Null	Present	Null
Jia W. *et al*.	2016	151 (33 + 118)	93 (32 + 61)	Reference	1.88 (1.01–3.47)		
Chen J.B. *et al*.	2016			Reference	0.82 (0.49–1.36)	Reference	0.78 (0.45–1.36)
Xiao H.L. *et al*.	2016			15.73	18.55		
		163 (80 + 83)	99 (63 + 36)	Reference	0.40 (0.23–0.69)		
Liu K. *et al*.	2015			35.16 ± 1.72	35.19 ± 2.16		
		155 (105 + 50)	107 (66 + 41)	Reference	0.85 (0.50–1.45)		
Wu G. *et al*.	2015	168 (68 + 100)	114 (55 + 59)	Reference	1.24 (0.74–2.11)		
Joerger M. *et al*.	2012			10.2 (7.3–11.5)	10.2 (8.2–15.7)	6.3 (4.9–7.6)	5.6 (4.5–6.8)
		80 (20 + 60)	57 (22 + 35)	Reference	1.13 (0.77–1.64)	Reference	0.97 (0.69–1.38)
Ada A.O. *et al*.	2010			Reference	0.91 (0.51–1.61)		
Kalikaki A. *et al*.	2009			10.2 (7.4–13.0)	11.3 (9.1–13.6)		
		72 (23 + 49)	42 (13 + 29)	Reference	1.2 (0.79–1.96)		
Li Q.Y. *et al*.	2014	45 (13 + 32)	44 (13 + 31)				
Mao Y. *et al*.	2007	31 (10 + 21)	28 (12 + 16)				
Sweeney C. *et al*.	2003			Reference	0.96–1.94		
Gonlugur U. *et al*.	2006			9.8 ± 1.1	11.7 ± 1.7		
Sreeja L. *et al*.	2008			31	16		
				Reference	1.2 (0.684–2.373)		
Ruano-Ravina A. *et al*.	2014			9.4 (8.2–10.6)	8.7 (6.0–11.4)		
				Reference	1.18 (0.72–1.91)		
Li W. *et al*.	2012	22 (8 + 14)	36 (25 + 11)	Reference	1.07 (0.70, 1.63)		
Li W. *et al*.	2008	57 (28 + 29)	84 (53 + 21)				
Jia W. *et al*.	2016	111 (28 + 83)	133 (37 + 96)	Reference	1.14 (0.62–2.11)		
Chen J.B. *et al*.	2016			Reference	0.81 (0.49–1.34)	Reference	0.67 (0.39–1.17)
Xiao H.L. *et al*.	2016			16.76	17.29		
		145 (77 + 68)	117 (66 + 51)	Reference	0.84 (0.49–1.43)		
Liu K. *et al*.	2015			34.81 ± 1.83	35.58 ± 2.00		
		141 (95 + 46)	121 (76 + 45)	Reference	0.88 (0.52–1.49)		
Wu G. *et al*.	2015	161 (69 + 92)	121 (54 + 67)	Reference	0.78 (0.47–1.31)		
Ada A.O. *et al*.	2010			Reference	1.18 (0.61–2.26)		
Kalikaki A. *et al*.	2009			11.3 (9.1–13.6)	4.3 (1.0–7.5)		
		106 (33 + 73)	6 (2 + 4)	Reference	1.2 (0.43–3.36)		
Sweeney C. *et al*.	2003			Reference	0.80–2.03		
Gonlugur U. *et al*.	2006			12.0 ± 1.6	8.9 ± 1.0		
Sreeja L. *et al*.	2008			23	14		
				Reference	2.1 (1.158–4.116)		
Ruano-Ravina A. *et al*.	2014			9.8 (8.0–11.5)	6.590.3–12.7)		
				Reference	1.48 (0.84–2.60)		

HR: hazard ratio; MST, median survival time (months); PFS, progression-free survival (months); ORR: objective response rate.

### Objective response rate of *GSTP1* Ile105Val genetic polymorphism

**Figure 2:**
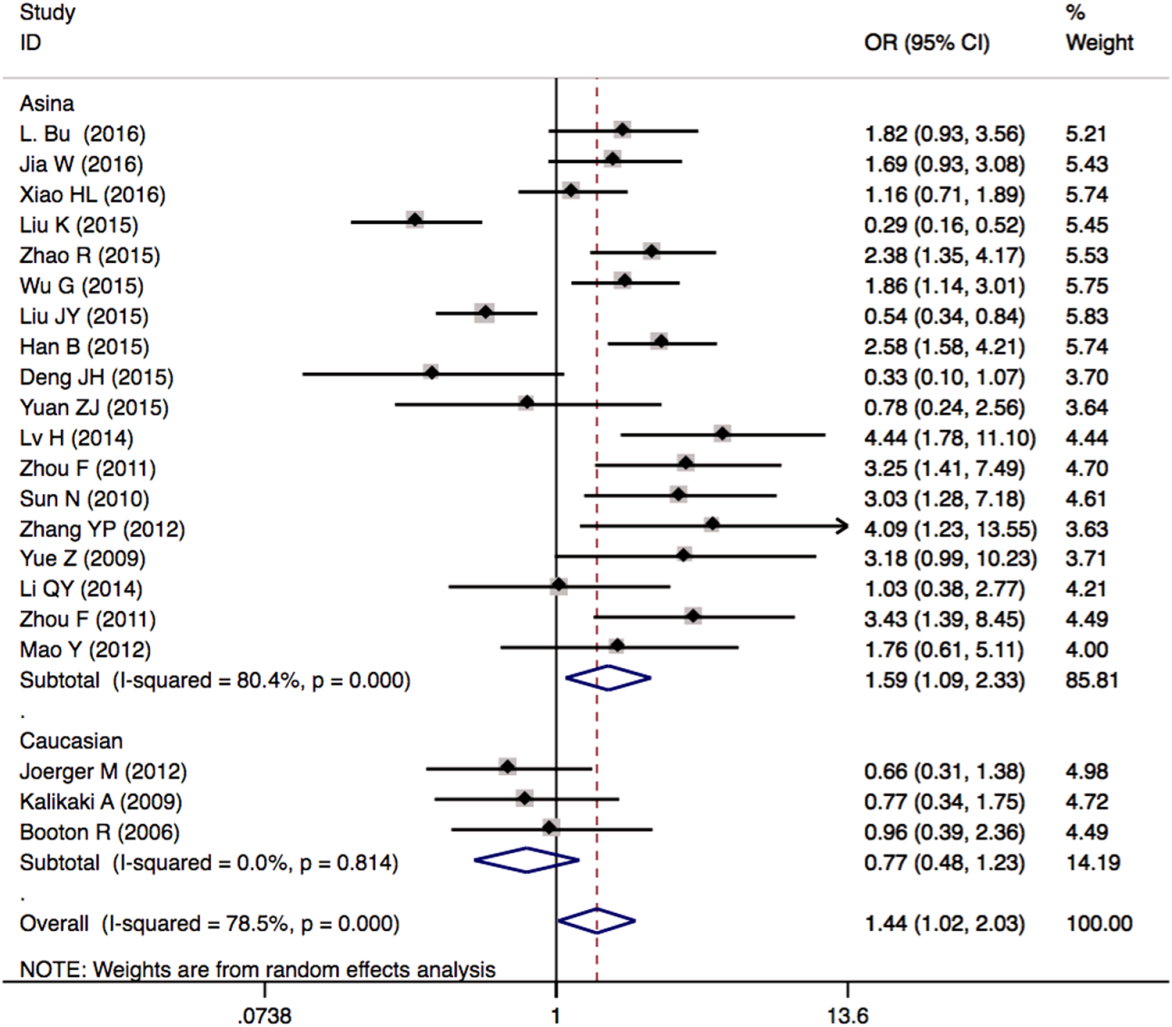
Forest plots of ORR in NSCLC patients treated with platinum-based chemotherapy by the *GSTP1* IIe105Val polymorphism (IIe/Val + Val/Val *vs*. IIe/IIe). Odds ratios (ORs) (and its 95% confidence interval (CI)) of objective response rate (ORR) stratified by ethnicity for *GSTP1* IIe105Val IIe/Val + Val/Val *vs*. IIe/IIe.

There were 21 publications including a total of 3200 patients enrolled for comparing the ORR in *GSTP1* Ile105Val different genotypic patients. The results showed that there was a statistically significant association between the *GSTP1* Ile105Val polymorphism and the ORR under dominant model (IIe/Val + Val/Val *vs*. IIe/IIe: odds ratio (OR) = 1.437, 95% confidence intervals (CIs), 1.019–2.027, *P* = 0.039). Subgroup analyses by ethnicity suggested that, for the Asian group, the association was significant (OR = 1.592 (1.087–2.332), *P* = 0.017); for the Caucasian group, the association was not significant (OR = 0.767 (0.479–1.228), *P* = 0.269) (Table 4 and Fig. 2). Moreover, we also carried out the subgroup analyses based on the evaluation criterion, genotyping method, and quality score. The results were shown in Figure S1 and Table S1. It implied that the contribution of *GSTP1* Ile105Val genetic polymorphism to the ORR of platinum-based chemotherapy has a manner of racial differences. Asian NSCLC patients (but not Caucasian NSCLC patients) bearing the favorable *GSTP1* IIe105Val + Val105Val genotypes were more likely to have better response rates to platinum-based chemotherapy compared to those with the unfavorable IIe105IIe genotype.

**Table 4:** Meta-analysis of the association between *GSTP1* IIe105Val polymorphism and platinum-based chemotherapy in objective response rate, overall survival and median progression-free survival for NSCLC patients.

Genetic comparisons	No. of studies	Study groups	Test of association			Model	Test of heterogeneity			Tau-squared
			OR/HR (95% CI)	Z	*P*-value		χ2	*P*-value	I^2^ (%)	
**Objective response rate (OR)**										
IIe/Val + Val/Val *vs*. IIe/IIe	21	Overall	**1.437 (1.019–2.027**)	2.07	**0.039**	R	93.05	<0.001	78.50%	0.4739
	3	Asian	**1.592 (1.087–2.332**)	2.39	**0.017**	R	86.54	<0.001	80.40%	0.5106
	18	Caucasian	0.767 (0.479–1.228)	1.10	0.269	R	0.41	0.814	0	0
Val/Val *vs*. IIe/IIe	14	Overall	1.374 (0.670–2.817)	0.87	0.385	R	78.21	<0.001	83.40%	1.3815
	12	Asian	1.645 (0.740–3.660)	1.22	0.222	R	74.25	<0.001	85.20%	1.4886
	2	Caucasian	0.495 (0.192–1.275)	1.46	0.145	R	0.31	0.578	0	0
IIe/Val *vs*. IIe/IIe	14	Overall	1.270 (0.920–1.754)	1.45	0.146	R	37.15	<0.001	68.90%	0.2304
	12	Asian	1.335 (0.932–1.912)	1.58	0.115	R	35.35	<0.001	65.00%	0.2591
	2	Caucasian	0.886 (0.481–1.630)	0.39	0.697	R	0.45	0.5	0.00%	0
Val/Val *vs*. IIe/IIe + IIe/Val	14	Overall	1.230 (0.687–2.202)	0.70	0.485	R	54.59	<0.001	77.50%	0.814
	12	Asian	1.431 (0.750–2.729)	1.09	0.276	R	0.12	<0.001	79.80%	0.8743
	2	Caucasian	0.515 (0.210–1.263)	1.45	0.147	R	57.89	0.724	0.00%	0
**Overall survival (HR)**										
IIe/Val *vs*. IIe/IIe	15	Overall	0.972 (0.798–1.184)	0.28	0.78	R	29.03	0.01	51.80%	0.0704
	10	Asian	0.867 (0.644–1.167)	0.94	0.345	R	21.03	0.012	57.20%	0.1238
	5	Caucasian	1.146 (0.940–1.397)	1.34	0.179	R	4.78	0.31	16.40%	0.0088
Val/Val *vs*. IIe/IIe	15	Overall	0.772 (0.504–1.182)	1.19	0.234	R	67.71	<0.001	79.30%	0.516
	10	Asian	0.559 (0.280–1.116)	1.65	0.099	R	58.74	<0.001	84.70%	0.9875
	5	Caucasian	1.121 (0.866–1.452)	0.87	0.384	R	3.11	0.54	0.00%	0
Val/Val + IIe/Val *vs*. IIe/IIe	2	Overall/Caucasian	0.833 (0.296–2.347)	0.35	0.729	R	7.32	0.007	86.30%	0.483
**PFS (HR)**										
IIe/Val *vs*. IIe/IIe	2	Overall	0.728 (0.207–2.566)	0.49	0.622	R	9.53	0.002	89.50%	0.7412
Val/Val *vs*. IIe/IIe	2	Overall	0.511 (0.049–5.317)	0.56	0.574	R	21.78	<0.001	95.40%	2.7277

OR, odds ratio; HR: hazard ratio; CI, confidence interval; vs., versus; F, fixed effect model; R, random effect model.

### Overall survival of *GSTP1* Ile105Val genetic polymorphism

There were 15 publications including a total of 4276 patients enrolled for comparing the overall survival rates in *GSTP1* Ile105Val different genotypic patients. The results showed that there were no statistically significant associations between the *GSTP1* Ile105Val polymorphism and OS under any genetic models (IIe/Val *vs*. IIe/IIe: OR = 0.972 (0.798–1.184), *P* = 0.78; Val/Val *vs*. IIe/IIe: OR = 0.772 (0.504–1.182), *P* = 0.234; Val/Val + IIe/Val *vs*. IIe/IIe: OR = 0.833 (0.296–2.347), *P* = 0.729) (Table 4). For the Asian group or the Caucasian group, there were no significant associations between the *GSTP1* Ile105Val polymorphism and OS under any genetic models (Table 4).

### Median progression-free survival of *GSTP1* genetic polymorphisms

There were 2 publications including a total of 430 patients enrolled for comparing the median progression-free survival rates in *GSTP1* Ile105Val different genotypic patients. The results showed that there were no statistically significant associations between the *GSTP1* Ile105Val polymorphism and PFS under any genetic models (IIe/Val *vs*. IIe/IIe: OR = 0.728 (0.207–2.566), *P* = 0.622; Val/Val *vs*. IIe/IIe: OR = 0.511 (0.049–5.317), *P* = 0.574) (Table 4).

### Objective response rate of *GSTM1* and *GSTT1* null or present genetic polymorphism

**Figure 3:**
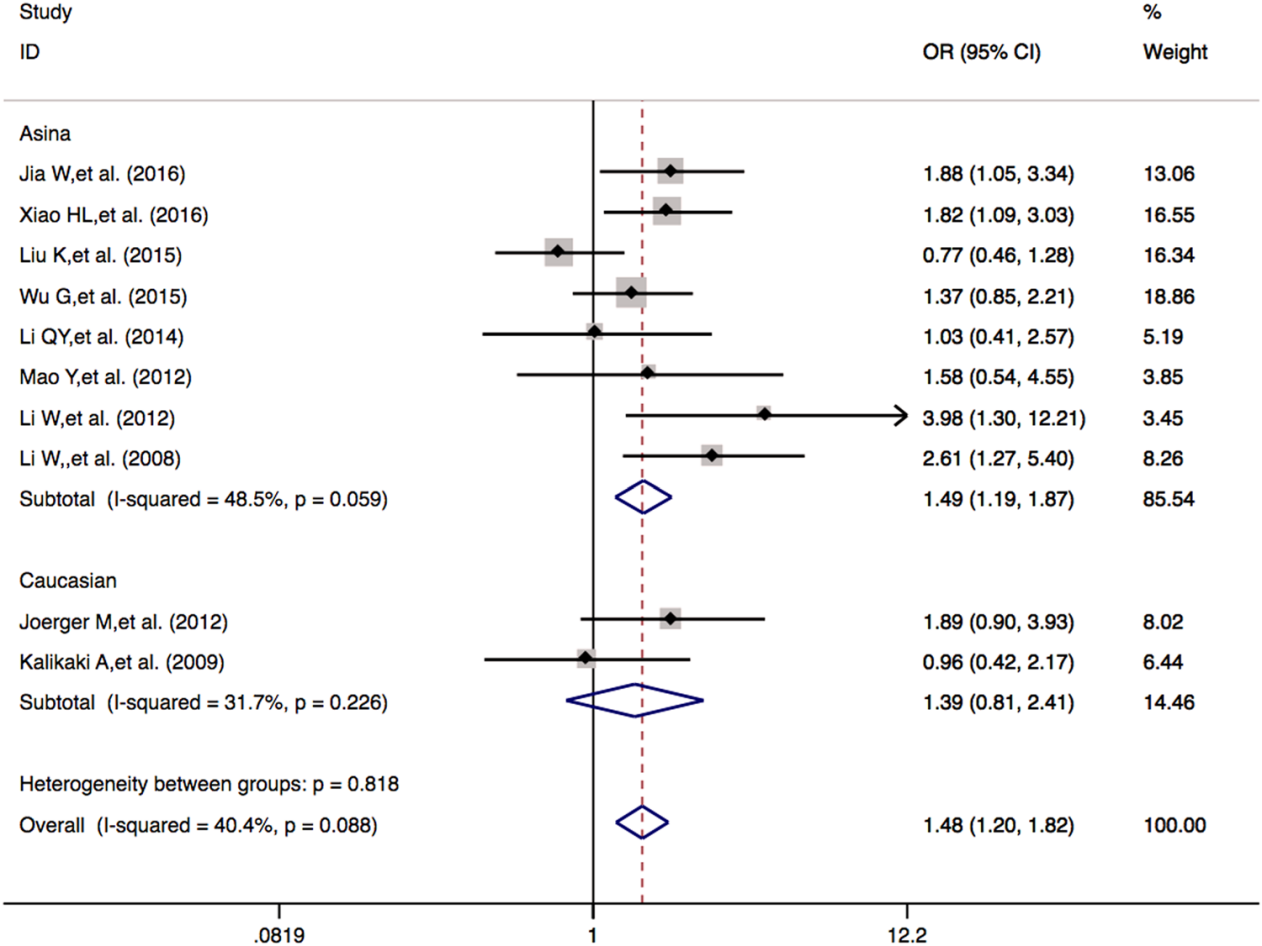
Forest plots of ORR in NSCLC patients treated with platinum-based chemotherapy by the *GSTM1* null or present polymorphism (null *vs*. present). Odds ratios (ORs) (and its 95% confidence interval (CI)) of objective response rate (ORR) stratified by ethnicity for the *GSTM1* null or present polymorphism IIe/Val + Val/Val *vs*. IIe/IIe.

There were 10 publications including a total of 1638 patients enrolled for comparing the objective response rates in the *GSTM1* null or present genotypic patients. The results showed that there were statistically significant associations between the *GSTM1* null or present polymorphism and ORR (null *vs*. present: OR = 1.478 (1.200–1.820), *P* < 0.001). Subgroup analyses by ethnicity suggested that, for the Asian group, the association was significant (OR = 1.493 (1.192–1.870), *P* < 0.001); for the Caucasian group, the association was not significant (OR = 1.393 (0.806–2.408), *P* = 0.236) (Table 5 and Fig. 3). The Asian NSCLC patients bearing the favorable *GSTM1* null genotype were more likely to have better response rates to platinum-based chemotherapy compared to those with the unfavorable *GSTM1* present genotype.

**Table 5:** Meta-analysis of the association between *GSTM1* and *GSTT1* polymorphisms and platinum-based chemotherapy in objective response rate, overall survival for NSCLC patients.

*GSTM1* (Null vs. Present)	No. of studies	Study groups	Test of association			Model	Test of heterogeneity			Tau-squared
Genetic comparisons			OR/HR (95% CI)	Z	*P*-value		χ2	P-value	I^2^ (%)	
Objective response rate (OR)	10	Overall	**1.478** (**1.200–1.820)**	3.68	**<0.001**	F	15.1	0.088	40.40%	—
	8	Asian	**1.493** (**1.192–1.870)**	0.368	**<0.001**	F	13.58	0.059	48.50%	—
	2	Caucasian	1.393 (0.806–2.408)	1.19	0.236	F	1.46	0.226	31.70%	—
Overall survival (HR)	12	Overall	1.054 (0.870–1.277)	0.53	0.593	R	20.41	0.04	46.10%	0.154
	6	Asian	0.936 (0.640–1.369)	0.34	0.732	R	15.98	0.007	68.70%	0.05
	6	Caucasian	1.190 (0.990–1.429)	1.86	0.063	R	1.53	0.91	0.00%	0
PFS/HR	2	Overall	0.912 (0.680–1.224)	0.61	0.539	F	0.43	0.513	0.00%	—
Objective response rate (OR)	5	Overall	1.035 (0.805–1.331)	0.27	0.8	F	1.12	0.891	0.00%	—
	4	Asian	1.033 (0.802–1.332)	0.25	0.91	F	1.12	0.773	0.00%	—
	1	Caucasian	1.106 (0.193–6.342)	0.11	0.79	F	—	—	—	—
Overall survival (HR)	10	Overall	1.076 (0.899–1.288)	0.8	0.424	F	10.3	0.327	12.60%	—
	5	Asian	0.867 (0.683–1.101)	1.17	0.242	F	1.02	0.907	0.00%	—
	5	Caucasian	**1.423** (**1.084–1.869)**	2.54	**0.011**	F	2.08	0.72	0.00%	—

OR, odds ratio; HR: hazard ratio; CI, confidence interval; vs., versus; F, fixed effect model; R, random effect model.

There were 5 publications including a total of 1162 patients enrolled for comparing the objective response rate in the *GSTT1* null or present genotypic patients. The results showed that there were no statistically significant associations between the *GSTT1* null or present polymorphism and ORR (null *vs*. present: OR = 1.035 (0.805–1.331), *P* = 0.80). Subgroup analyses by ethnicity suggested that, for the Asian group, the association was also not significant (OR = 1.033 (0.802–1.332), *P* = 0.91); for the Caucasian group, the association was not significant (OR = 1.106 (0.193–6.342), *P* = 0.79) (Table 5).

### Overall survival and median progression-free survival of *GSTM1* and *GSTT1* null or present genetic polymorphism

There were 12 publications including a total of 2638 patients enrolled for comparing the overall survival rates in the *GSTM1* null or present genotypic patients. The results showed that there were no statistically significant associations between the *GSTM1* null or present and OS (null *vs*. present: OR = 1.054 (0.870–1.277), *P* = 0.593). For the Asian group or Caucasian group, there were no significant associations between the *GSTM1* null or present genotype and OS (Table 5).

There were 2 publications including a total of 430 patients enrolled for comparing the median progression-free survival rates in the *GSTM1* null or present genotypic patients. The results showed that there were no statistically significant associations between the *GSTM1* null or present and PFS (null *vs*. present: OR = 0.912 (0.680–1.224), *P* = 0.539) (Table 5).

**Figure 4:**
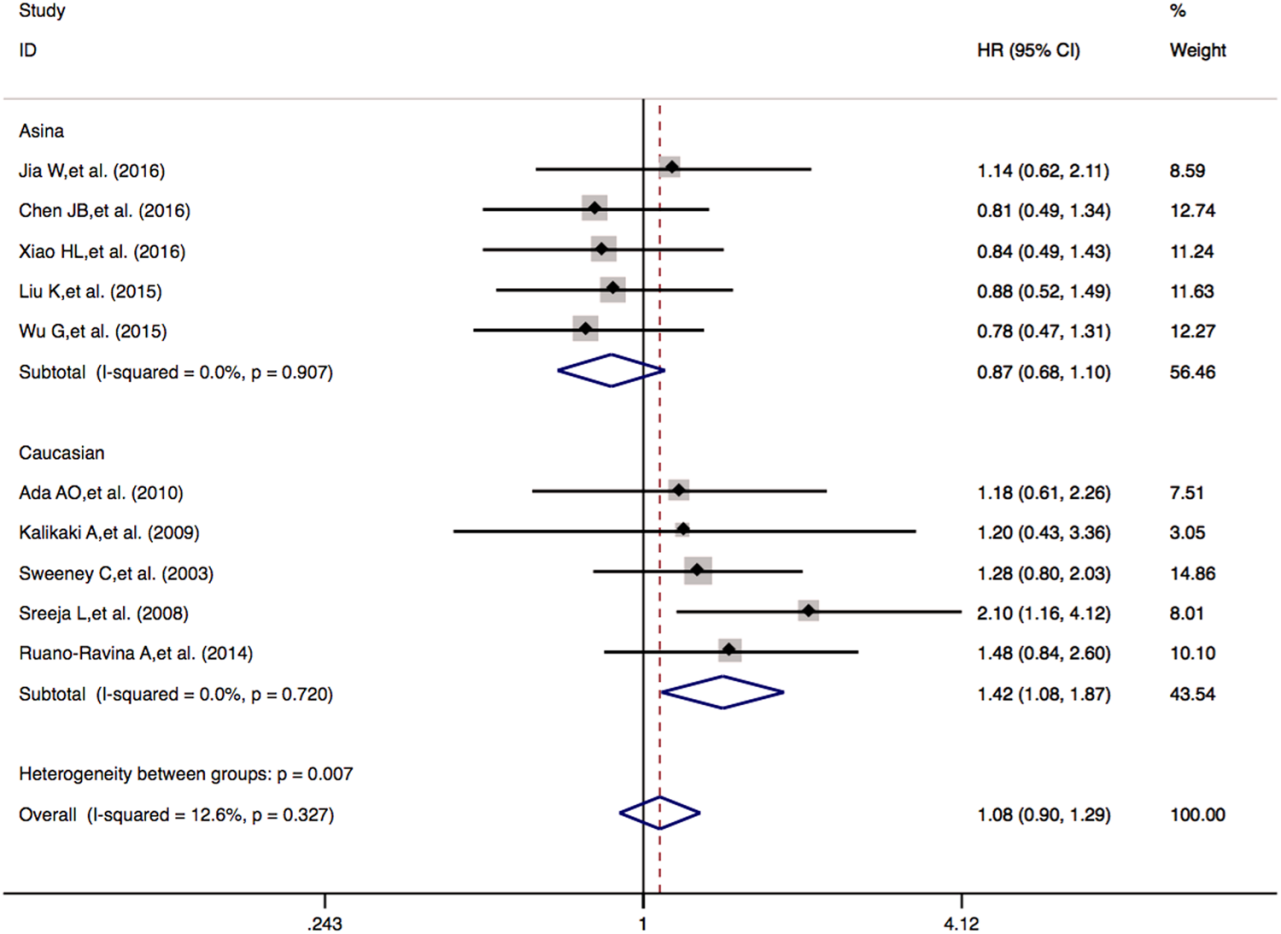
Forest plots of OS in NSCLC patients treated with platinum-based chemotherapy by the *GSTM1* null or present polymorphism (null *vs*. present). Hazard ratios (HRs) (and its 95% confidence interval (CI)) of overall survival (OS) stratified by ethnicity for the *GSTM1* null or present polymorphism null *vs*. present.

There were 10 publications including a total of 2275 patients enrolled for comparing the overall survival rates in the *GSTT1* null or present genotypic patients The results showed that there were no statistically significant associations between the *GSTT1* null or present and OS (null *vs*. present: OR = 1.076 (0.899–1.288), *P* = 0.424). For the Asian group, there was no significant association (null *vs*. present: OR = 0.867 (0.683–1.101), *P* = 0.242). However, for the Caucasian group, there was significant association between the *GSTT1* null or present genotype and OS (null *vs*. present: OR = 1.423 (1.084–1.869), *P* = 0.011) (Table 5 and Fig. 4). The results suggested that the Caucasian lung cancer patients bearing the *GSTT1* null genotype might be more closely associated with shorter survival time and higher risks of death than the *GSTT1* present patients.

### Publication bias and sensitivity analysis

**Figure 5:**
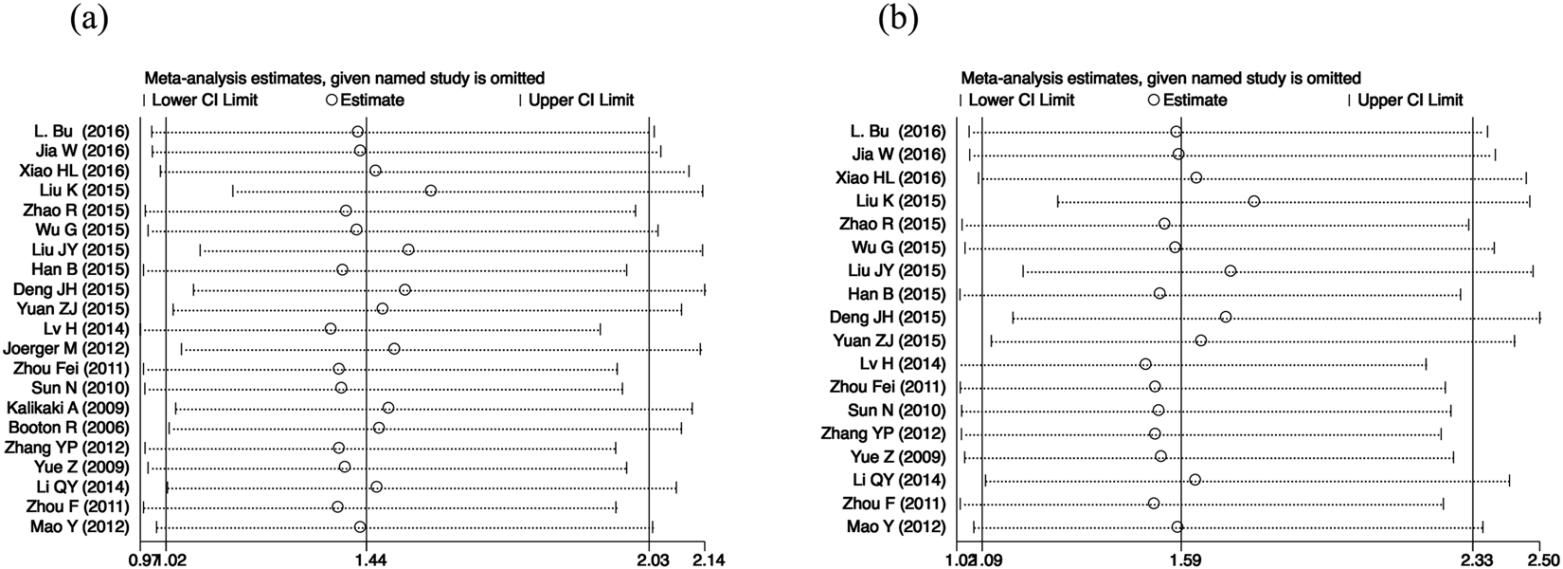
The sensitivity analysis of pooling ORs of ORR in NSCLC patients treated with platinum-based chemotherapy by the *GSTP1* IIe105Val polymorphism (IIe/Val + Val/Val *vs*. IIe/IIe). (**a**) in overall patients, (**b**) in Asian patients.

Publication bias was examined by Egger’s test and Begg’s test. As shown in Figure S1, Begg’s funnel plots and Egger’s funnel plots under the *GSTP1* IIe105Val dominant model (IIe/Val + Val/Val *vs*. IIe/IIe) appear approximately symmetrical and show no publication bias (*P* = 0.833, *P* = 0.467, respectively). As for the *GSTM1* null or present genetic polymorphism, the shapes of the Begg’s funnel plots and Egger’s funnel plots seem approximately symmetrical and show no publication bias (*P* = 0.592, *P* = 0.399, respectively). The shapes of the Begg’s funnel plots and Egger’s funnel plots of *GSTT1* null or present genetic polymorphisms seem not symmetrical and show publication bias (*P* = 0.007, *P* = 0.002, respectively, Figure S3). After being divided into two groups according to ethnicity, the shapes of the Begg’s funnel plots and Egger’s funnel plots of *GSTT1* null or present genetic polymorphism in the Asian population have publication bias (*P* = 0.027, *P* = 0.002, respectively, Figure S3). However, there is no publication bias of *GSTT1* null or present genetic polymorphism in the Caucasian population (*P* = 0.221, *P* = 0.385, respectively, Figure S3). Sensitivity analysis results show that changing the effect models had no significant effects on the pooled OR, HR and the final strength of the association between *GSTP1* IIe105Val, *GSTM1* and *GSTT1* null or present genetic polymorphisms and the clinical outcome of platinum-based chemotherapy to NSCLC patients. Moreover, Fig. 5 show the results of sensitivity analysis regarding ORR of *GSTP1* IIe105Val dominant model (IIe/Val + Val/Val *vs*. IIe/IIe) in overall population or Asian population. We found that excluded studies did not influence the overall effective size in the Asian population.

## Discussion

In this meta-analysis, results show that *GSTP1* IIe105Val IIe/Val and Val/Val genotypic Asian NSCLC patients were more likely to have better response rates compared to *GSTP1* IIe105Val IIe/IIe patients. The Asian NSCLC patients bearing the favorable *GSTM1* null genotype were more likely to have better response rates to platinum-based chemotherapy compared to those patients with the unfavorable *GSTM1* present genotype. Caucasian NSCLC patients bearing *GSTT1* null genotype might be more closely associated with shorter survival time and higher risks of death than the *GSTT1* present patients. Herein, we suggested that the *GSTP1* IIe105Val, *GSTM1* and *GSTT1* null or present genetic polymorphisms might be predictive factors for the efficacy of platinum-based chemotherapy to NSCLC patients.

The platinum-based chemotherapy is the standard first-line and effective therapies for NSCLC patients, especially for advanced cancer. However, the efficacy of the platinum-based chemotherapy varies wildly among patients. Previous studies provide the evidences that genetic variants of genes involved in the detoxification and DNA repair pathways including *GSTP1*, *GSTM1*, *GSTT1*, *ERCC1*, *XPD*, *XPG*, *XRCC1* may influence the anti-cancer efficacy of platinum-based chemotherapy^14, 29, 38, 41–45^. However, their results were inconsistent and need meta-analysis and further confirmation. *GSTP1*, *GSTM1*, and *GSTT1* are three genes of human glutathione S-transferases (GSTs) super family members, which have crucial roles in metabolizing most cytotoxic cancer chemotherapeutic agents such as the platinum detoxification^46, 47^. One nonsynonymous polymorphism occurring in *GSTP1* (IIe105Val) in exon 5 and allelic deletions in the *GSTM1* and *GSTT1* variants are associated with the lower substrate specific catalytic activity and the reduced enzyme activity, thus lowering the intracellular concentration of chemotherapeutic agents^16, 48^. Therefore, the patients who suffer the favorable *GSTP1* IIe105Val and Val105Val genotypes may display a reduced ability to detoxify drug metabolites, thus promoting better response rates to platinum-based chemotherapy. Allelic deletions in the *GSTM1* and *GSTT1* genotypes are associated with reduced enzyme activity thus they could be predictive factors of the efficacy of platinum-based chemotherapy.

Published data have indicated that the *GSTP1* IIe105Val variant might be associated with the efficacy of platinum-based chemotherapy in lung cancer patient^16, 19–21, 23, 24, 27, 29^. However, there were also some negative results about *GSTP1* IIe105Val variant^15, 22, 28, 31, 49^. In our meta-analysis, we found the significant association between *GSTP1* IIe105Val dominant model and ORR of Asian NSCLC patients treated with platinum-based chemotherapy (Table 4). Because of the heterogeneity, random model were used to pool the OR of ORR of overall patients and Asian patients. Moreover, we carried out the sensitivity analysis and results showed that changing the effect models had no significant effects on the pooled OR of ORR and the final strength of the association between *GSTP1* IIe105Val and ORR of Asian NSCLC patients treated with platinum-based chemotherapy. Moreover, excluded studies did not influence the overall effective size in Asian population (Fig. 5). Lung cancer is a kind of complicated illness and different ethnicities have different genetic backgrounds, which may affect the anti-cancer therapeutic outcome of platinum-based chemotherapy. Herein, we evaluated the relationship of *GSTP1*, *GSTM1*, and *GSTT1* variants and the efficacy of platinum-based chemotherapy stratified by different ethnicities. There were two ethnicities enrolled in our studies: Asian and Caucasian. Our analysis found no significant association on *GSTP1* IIe105Val variant and clinical outcome of platinum-based chemotherapy in Caucasian patients (Table 4). It implies that the ethnic difference also influence the contribution of the *GSTP1* IIe105Val variant to the variation of clinical outcomes of platinum-based chemotherapy. Therefore, the ethnic factor should be considered and weighed with caution when we drawn the conclusions from our meta-analysis. Ethnic individual platinum-based chemotherapy treatment for NSCLC patients should be conducted in the future.

*GSTM1* and *GSTT1* are located on chromosome 1p13.3 and 22q11.2. Homozygous of *GSTM1* and *GSTT1* null genotypes lead to an absence of enzymatic activity^50–52^. The relationships of *GSTM1* and *GSTT1* genotypes and the survival rates in lung cancer are revealed to be quite conflicting also. Several studies have not found significant associations^21, 23, 49, 53, 54^, while others have found significant associations^20, 22, 25, 34^. Our meta-analysis suggests that the lung cancer patients bearing the favorable *GSTM1* null genotype were more likely to have better response rates to platinum-based chemotherapy compared to those with the unfavorable *GSTM1* present genotype in Asian patients, but not in Caucasian patients (Table 5 and Fig. 3). In Caucasian group, there was significant association between the *GSTT1* null or present genotype and overall survival (null *vs*. present: OR = 1.423 (1.084–1.869), *P* = 0.011) (Table 5 and Fig. 4).

Important things that cannot be ignored in meta-analysis are heterogeneity and publication bias. We carried out the Q test and I^2^ statistics to test the significance of heterogeneity. There were obvious heterogeneities in pooled ORR, OS and PFS of *GSTP1* IIe105Val variant patients (Table 4). Therefore the random model was used. In order to find out the source of heterogeneity, we conducted subgroup analysis by ethnicity. However, after the subgroup analysis by ethnicity, there were still heterogeneities in Asian group even when clinical outcome were pooled (Table 4). In contrast, in the Caucasian group, there were no heterogeneities, indicating the heterogeneity could be partly accounted for by the genetic distribution in different ethnicities. In addition, the inconsistency of these studies about Asian patients may be due to the source of the patients, disease condition, publication qualities or other clinical issues. Further large sample multi-center studies are needed. In order to draw more cautious conclusion on *GSTP1* IIe105Val, we also carried out the sensitivity analysis. Results showed that changing the effect models had no significant effects and excluding some studies did not influence the overall effective size in pooled OR value of ORR in the Asian population (Fig. 5). We used Egger’s test and Begg’s test to analyze publication bias. There was no publication bias in *GSTP1* IIe105Val and *GSTM1* null or present genetic polymorphism on clinical outcome of platinum-based chemotherapy (Figure S2). We have seen the publication bias in *GSTT1* null or present variant on clinical outcome of platinum-based chemotherapy (Egger’s test *P* = 0.002, Begg’s test *P* = 0.007, Figure S3). After the subgroup analysis by race, the publication bias has disappeared in the Caucasian group (Egger’s test *P* = 0.221, Begg’s test *P* = 0.385) but not in the Asian population (Egger’s test *P* = 0.027, Begg’s test *P* = 0.002, Figure S3). Herein, we could drawn the conclusion that the Caucasian lung cancer patients bearing *GSTT1* null genotype might be more closely associated with shorter survival time and higher risks of death than the *GSTT1* present patients and there was no publication bias in this meta-analysis about *GSTT1* null genotype and survival time in the Caucasian population.

Our meta-analysis pooled ORR, OS and PFS of NSCLC patients treated with platinum-based chemotherapy harboring different *GSTM1* and *GSTT1* null genotypes.

After our precise and comprehensive assessment of the update system review and meta-analysis, pooled ORR, OS and PFS enrolled a total of 5712 NSCLC patients treated with platinum-based chemotherapy in our comprehensive and systematic evaluation of efficacy. We found that *GSTP1* IIe105Val, *GSTM1* and *GSTT1* null genetic polymorphisms might be predictive factors for the efficacy of platinum-based chemotherapy to NSCLC patients.

Previously, there were two meta-analyses that revealed the *GSTP1* IIe105Val, *GSTM1* null genetic polymorphisms and the efficacy of platinum-based chemotherapy in NSCLC patients and no meta-analysis about *GSTT1* null genetic polymorphisms and the efficacy of platinum-based chemotherapy in NSCLC patients^17, 40^. These results from the two meta-analyses seem conflicting rather than conclusive for each other. The different studies enrolled in their analysis may possibly bias the conclusions. In our meta-analysis, we systematically enrolled all available up-to-date studies related with *GSTP1* IIe105Val, *GSTM1* and *GSTT1* null genetic polymorphisms and the efficacy of platinum-based chemotherapy to NSCLC patients.

Our updated meta-analysis enrolled 29 publications including 5414 NSCLC patients harboring *GSTP1* IIe105Val variant, 16 publications including 3008 NSCLC patients harboring *GSTM1* null or present variant, 11 publications including 2356 NSCLC patients harboring *GSTT1* null or present variant, which are several times more than the previous two meta-analyses. Therefore, our meta-analysis is more precise and reliable in predicting the role of *GSTP1*, *GSTM1* and *GSTT1* polymorphisms on the clinical outcome of platinum-based chemotherapy in NSCLC patients.

Despite our efforts to conduct a comprehensive and accurate meta-analysis, it still has several limitations, which should be taken into account in interpreting the existing results. First of all, the sample sizes and numbers of enrolled studies in our meta-analysis are still limited, especially in the subgroup analysis and single studies (range from 59 to 420). Only 9 publications of patients were of Caucasian populations, which also limited the generalizability to other ethnic populations. Some indicators such as TTP or PFS may have been undervalued in analysis because of the limited numbers of enrolled studies. The second limitation is the significant heterogeneity between studies in pooled analysis for *GSTP1*, although it is unlikely to influence the final conclusion after other analyses are carried out, such as stratified analyses by race, sensitivity analysis, and the changes of analysis models. Thirdly, the variation in the patients’ characteristics in each study, such as age, gender percentage, ethnicity, TNM staging, smoking history, specific anti-cancer drugs, chemotherapy regimens, test methods, may also influence the heterogeneity of studies and the final conclusions. Moreover, the quality of publications is still in need of further accurate and precise improvement.

## Conclusions

In conclusion, our meta-analysis indicates that the *GSTP1* IIe105Val, *GSTM1* and *GSTT1* null or present genetic polymorphisms might be predictive factors for the efficacy of platinum-based chemotherapy to NSCLC patients. *GSTP1* IIe105Val IIe/Val and Val/Val genotypic NSCLC patients were more likely to have better response rates compared to those IIe/IIe genotypic Asian patients. The lung cancer patients bearing the favorable *GSTM1* null genotype were more likely to have better response rates to platinum-based chemotherapy compared to those with the unfavorable *GSTM1* present genotype, especially in Asian patients. Caucasian lung cancer patients bearing *GSTT1* null genotype might be more closely associated with shorter survival time and higher risks of death than the *GSTT1* present patients. In the future, well-designed pharmacogenetic studies with multi-center, multi-ethnic and large sample sizes are needed to draw a more accurate and robust conclusion.

## Materials and Methods

### Study review and selection

We reviewed the databases including PubMed, EMBASE, Web of Science, Wanfang and CNKI to 14 Oct. 2016. The searching strategy was “GSTP1 or GSTP1 glutathione S-transferase pi 1”, “GSTM1 or glutathione S-transferase mu 1”, “GSTT1 or glutathione S-transferase theta 1”, “lung cancer or carcinoma or tumor”, “SNPs or genetic polymorphisms or variations”, “pharmacogenomics”, “platinum or cisplatin or carboplatin or nedaplatin, lbaplatin, oxaliplatin” and “chemotherapy” Dr. Qiang Qu and Dr. Huan Ye reviewed all relevant articles to identify potential eligible studies.

### Inclusion and exclusion criteria

The inclusion criteria are: (1) NSCLC patients; (2) At least having one of *GSTP1* IIe105Val, *GSTM1* and *GSTT1* null or present genetic polymorphisms data; (3) At least having one clinical indicator (ORR, OS, PFS, TTP, OR and HR with corresponding to 95% CIs); (4) Treatments having platinum-based chemotherapy. A study was excluded if any of the following exclusion criteria applies: (1) having no relevance to cancer and clinical patients; (2) having no variants information or having no clinical indicators; (3) Involving just in animals or cells; or being a review, or being an abstract with no data. Different opinions on study selections were solved in a discussion by all authors.

### Data collection and quality assessment

Two investigators (Dr. Qiang Qu and Dr. Huan Ye) independently extracted data from eligible studies. Different opinions on study selections were solved by all author’s discussion. The data were extracted as follows: authors’ names, sex, smoking status, ethnicities (Asian and Caucasian), clinical stage, evaluation criterion, genotyping methods, outcomes (ORR, OS, PFS, MST, TTP, OR and 95% CI), and the number of responders and non-responders in different genotypes. The QS for each study was also evaluated separately by two investigators (Dr. Jian Qu and Dr. Meiqin Shao) using previous methods^6^. According to the QS, every study has its score range from 0 to 24 reflecting cancer clinical stage, evaluation criteria, platinum combinations, genotyping methods, OS, PFS, MST, and sample size. The literature with QS ≤ 14 was considered low quality and the literature with QS > 14 was considered high quality.

Response evaluation criteria in solid tumors (RECISTC) or World Health Organization (WHO) were used to evaluate therapeutic efficacy including complete response (CR), partial response (PR), stable disease (SD) and progressive disease (PD). We evaluated the ORs and 95% CIs for the objective response rate (ORR) and no response after platinum-based chemotherapy (CR + PR *vs*. PD + SD). PRISMA checklist was used for our meta-analysis guideline^55^.

### Statistical analysis

We used STATA version 12 (Stata Corp, College Station, TX, USA) to carry out the meta-analysis. Heterogeneity was assessed by the Cochrane’s *Q*-statistic test and *I* ^*2*^ test. Random effect model was used in the analysis if *P* < 0.05 and *I* ^*2*^ > 50%, otherwise, a fixed effect model was chosen^56^. The significance of the pooled ORs was estimated using the Z-test. Publication bias was analyzed by Egger’s test and Begg’s test. Tests were two-sided and statistical significance was accepted at *P* < 0.05.

## Electronic supplementary material

Additional information
