# Supplementary material for: Predictive assessment in pharmacogenetics of Glutathione S-transferases genes on efficacy of platinum-based chemotherapy in non-small cell lung cancer patients
Source: Sci Rep. 2017 Jun 1;7:2670. doi: 10.1038/s41598-017-02833-7 (PMC5453955; doi:10.1038/s41598-017-02833-7)
Supplement: Supplementary file 1 — Additional information [file 41598_2017_2833_MOESM1_ESM.doc]

**Predictive assessment in pharmacogenetics of Glutathione S-transferases genes on efficacy of platinum-based chemotherapy in non-small cell lung cancer patients**

Huan Ye, Meiqin Shao, Xiaohong Shi, Lifeng Wu, Bing Xu, Qiang Qu, Jian Qu

**Additional information**

**Figure S1: Forest plots of ORR in NSCLC patients treated with platinum-based chemotherapy by the *GSTP1* IIe105Val polymorphism (IIe/Val + Val/Val vs. IIe/IIe).** Odds ratios (ORs) (and its 95% confidence interval (CI)) of objective response rate (ORR) were stratified by QS (a), evaluation criterion (b) and genotyping method (c) for *GSTP1* IIe105Val IIe/Val+Val/Val vs. IIe/IIe.

**Figure S2: Begg’s and Egger’s bias plot for publication bias test in the enrolled studies on the association between the *GSTP1* IIe105Val polymorphism and objective response rate of NSCLC patients treated with platinum-based chemotherapy under dominant model (IIe/Val+Val/Val vs. IIe/IIe).**

OR: odds ratio; SE: standard error.(a) Begg’s bias plot in overall patients. (b) Begg’s bias plot in Asian patients. (c) Egger’s bias plot in overall patients. (d) Egger’s bias plot in Asian patients.

**Figure S3: Begg’s and Egger’s bias plot for publication bias test in the enrolled studies on the association between the *GSTT1* null or present genetic polymorphism and overall survival of NSCLC patients treated with platinum-based chemotherapy.**

HR: hazards ratio; SE: standard error.(a) Begg’s bias plot in overall patients. (b) Begg’s bias plot in Asian patients. (c) Begg’s bias plot in Caucasian patients. (d) Egger’s bias plot in overall patients. (e) Egger’s bias plot in Asian patients. (f) Egger’s bias plot in Caucasian patients.

**Table S1: Meta-analysis of the association between *GSTP1* IIe/Val + Val/Val vs. IIe/IIe and platinum-based chemotherapy in objective response rate for NSCLC patients base on QS，evaluation criterion and genotyping method.**

| No. of studies | Study groups | Test of association | | |  | Test of heterogeneity | | |  |
| --- | --- | --- | --- | --- | --- | --- | --- | --- | --- |
|  |  | OR (95% CI) | Z | P-value | Model | χ2 | P-value | I^2^(%) | Tau-squared |
|  | QS |  |  |  |  |  |  |  |  |
| 6 | Low quality | 1.342(0.879-2.048) | 2.78 | 0.005 | R | 5.44 | 0.365 | 0.08 | 0.021 |
| 15 | High quality | 1.342(0.879-2.048) | 1.36 | 0.173 | R | 84.27 | <0.001 | 0.834 | 0.5537 |
|  | Evaluation criterion |  |  |  |  |  |  |  |  |
| 17 | RECIST | 1.234(0.851-1.790) | 1.11 | 0.266 | R | 78.71 | <0.001 | 0.797 | 0.4566 |
| 2 | WHO | 3.627(1.936-6.793) | 4.02 | <0.001 | R | 0.35 | 0.553 | 0 | 0 |
| 2 | NR | 2.301(1.047-5.057) | 2.07 | 0.038 | R | 0.54 | 0.464 | 0 | 0 |
|  | Genotyping method |  |  |  |  |  |  |  |  |
| 10 | PCR-RFLP | 1.283(0.797-2.065) | 1.03 | 0.305 | R | 59.6 | <0.001 | 0.849 | 0.485 |
| 7 | DNA sequencing | 1.124(0.610-2.073) | 0.37 | 0.708 | R | 17.69 | 0.007 | 0.661 | 0.4433 |
| 2 | TaqMan PCR | 4.308(2.080-8.920) | 3.93 | <0.001 | R | 0.01 | 0.915 | 0 | 0 |

OR, odds ratio; CI, confidence interval; R, random effect model; QS, quality score; PCR-RFLP, PCR-restriction fragment length polymorphism; RECIST, Response Evaluation Criteria in Solid Tumors; WHO, World Health Organization; NR: not reported.

**PRISMA flow diagram**

Studies after duplicates removed
(n = 254)

Studies screened
(n = 36)

Full-text articles assessed for eligibility (Studies = 31，Patients No=5712)

No detail data for meta- analysis(n = 5)

Studies identified through PubMed, EmBase, CNKI, Wangfang and Web of Science databases searching
(n =1185)

Excluded not relevant publications after review the titles and abstracts:

1. Irrelevant studies(n=162)

2. Meta-analysis(n=25)

3. Case report(n=3)

4. Basic research (n=28)

Studies included in *GSTM1* meta-analysis (Studies = 16，Patients No.= 3008)

Studies included in *GSTP1* meta-analysis
(Studies = 29，Patients No.=5414)

Studies included in *GSTT1* meta-analysis

(Studies = 11，Patients No.= 2356)
